# Predicting Patient Length of Stay in Australian Emergency Departments Using Data Mining

**DOI:** 10.3390/s22134968

**Published:** 2022-06-30

**Authors:** Sai Gayatri Gurazada, Shijia (Caddie) Gao, Frada Burstein, Paul Buntine

**Affiliations:** 1 Faculty of Information Technology, Monash University, Clayton, Melbourne, VIC 3800, Australia; sgur0006@student.monash.edu (S.G.G.); frada.burstein@monash.edu (F.B.); 2 Eastern Health Clinical School Monash University, Box Hill, Melbourne, VIC 3128, Australia; paul.buntine@easternhealth.org.au

**Keywords:** clinical decision support, data mining models, emergency department, length of stay, predictive data mining, Weka

## Abstract

Length of Stay (LOS) is an important performance metric in Australian Emergency Departments (EDs). Recent evidence suggests that an LOS in excess of 4 h may be associated with increased mortality, but despite this, the average LOS continues to remain greater than 4 h in many EDs. Previous studies have found that Data Mining (DM) can be used to help hospitals to manage this metric and there is continued research into identifying factors that cause delays in ED LOS. Despite this, there is still a lack of specific research into how DM could use these factors to manage ED LOS. This study adds to the emerging literature and offers evidence that it is possible to predict delays in ED LOS to offer Clinical Decision Support (CDS) by using DM. Sixteen potentially relevant factors that impact ED LOS were identified through a literature survey and subsequently used as predictors to create six Data Mining Models (DMMs). An extract based on the Victorian Emergency Minimum Dataset (VEMD) was used to obtain relevant patient details and the DMMs were implemented using the Weka Software. The DMMs implemented in this study were successful in identifying the factors that were most likely to cause ED LOS > 4 h and also identify their correlation. These DMMs can be used by hospitals, not only to identify risk factors in their EDs that could lead to ED LOS > 4 h, but also to monitor these factors over time.

## 1. Introduction

A continual year on year increase in demand for emergency services in many Australian EDs has created a challenging environment, with prolonged Emergency Department (ED) waiting times, delays in service, and a potential for a decline in the quality of services rendered [1,2]. As a result, patients frequently stay for longer durations of time in the ED than is optimal. Studies found that managing the patient Length of Stay (LOS) in the ED could be the key to improving the quality of emergency care offered in hospitals [3]. ED LOS can be defined as the time spent by a patient in the ED from the time of their arrival until they physically leave the ED or are admitted into the hospital [4]. An increase in in-hospital mortality has previously been observed to be associated with stays in excess of 4 h [5], but despite widespread attention to this metric, at least one-third of all ED presentations in Australian hospitals still report an LOS greater than 4 h [6]. An ED presentation can be defined as any individual who seeks treatment at an ED. This is used by Australian hospitals as a measure to count patients arriving at their EDs [7]. These delays in the EDs have been reported to occur due to the inefficiencies in the ED processes. This study aims to identify factors associated with an ED LOS greater than 4 h and to determine whether data mining (DM) techniques can be used for predictive modelling once these factors have been identified. Previous published literature has reported several human and organizational factors to be responsible for ED LOS exceeding 4 h [4,6]. Since processes in EDs vary throughout the world, this study only focuses on those factors relevant to Australian EDs [8,9,10]. Controlling these factors could be beneficial for managing ED LOS.

In recent years, the large volume of data being generated and collected by hospitals has contributed to the advancements in the applications of Data Mining (DM). This has led to significant breakthroughs in using DM as part of Clinical Decision Support (CDS). Although the value of applying DM techniques for CDS has been acknowledged, there is still limited research into its potential. The application of DM tools can help hospital managements manage ED LOS and allow them to implement strategies for meeting this metric.

The objective of this study is to build Data Mining Models (DMMs) for CDS using factors shown to be associated with delays in ED LOS in the previously published literature. These models could potentially be customized and used in hospitals to make evidence-based strategic decisions to improve their ED processes. Building these DMMs will be beneficial not only for identifying factors that impact ED LOS but also for establishing any correlation between them.

This study was conducted in collaboration with one of Melbourne’s largest healthcare providers (hereafter referred to as “Healthcare Service A”) with eight active locations and three EDs. All healthcare providers in Victoria, Australia (where Melbourne is the capital city) record administrative and clinical patient data for their ED presentations in the Victorian Emergency Minimum Dataset (VEMD) [11]. Data collection across all jurisdictions in Australia is aligned [12]. Hence, data collected in the VEMD are similar to data used in other Australian studies in the ED LOS context, and to basic administrative data such as that captured by the VEMD is highly representative of that captured by EDs throughout Australia [6,13]. A deidentified data extract containing all elements from VEMD plus additional administrative data were obtained from Healthcare Service A to be used in this study. To ensure its quality, the dataset was pre-processed before analysis. Both the pre-processing and data analysis were performed using the Weka software [14,15]. A total of six DMMs: Random Forest (RF), Naïve Bayes (NB), K-Nearest Neighbour (K-NN), J48 Decision Tree (DT), Logistic Regression (LR), and ZeroR were implemented in this study. The K-NN algorithm was implemented using the LazyIBK model available on Weka. Many studies in the healthcare DM context adopted the ROC curve, accuracy, precision, or f-measure as a measure of model performance [13,16,17,18]. Only accuracy and ROC were adopted as model performance measures in the Australian ED LOS context [6,13]. Hence, the performance of these models was compared primarily using the Receiver Operating Characteristic (ROC) value and model accuracy. The LazyIBK algorithm had the best performance out of the six models, with an accuracy of 74% and an ROC value of approximately 0.82. This study found that the quality of the data has a significant impact on the model performance.

The structure of this paper is outlined as the following. This paper firstly discusses the findings from a systematic literature survey which aims to identify the current applications of DM in the patient LOS context. This section also explores various factors that influence patient LOS and determine which of them are most relevant to ED LOS, particularly in the Australian ED context. Following this, the methodology of the research including dataset selection, data quality, data pre-processing, and data analysis are discussed. Next, the results from the data analysis are presented and discussed in comparison to similar studies. Finally, limitations of the study and recommendations for future work are discussed, along with a conclusion.

## 2. Literature Survey

**Figure 1:**
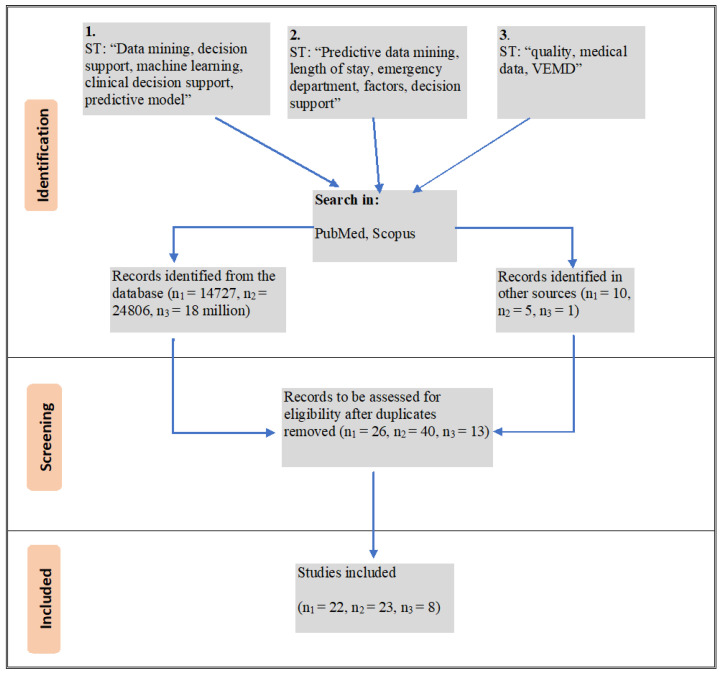
Systematic literature survey to identify suitable studies.

This section firstly discusses the general processes in an Australian ED followed by suitable DMMs that can be used in the ED LOS context. It also discusses the factors that are likely to risk delays in the ED as suggested by other studies. Both the DMMs and factors discussed in this section were identified by conducting a systematic literature survey based on their suitability to the Australian ED context. PubMed and Scopus were the primary databases used to identify literature for this study. Additionally, some other government websites were used when necessary. PubMed and Scopus were the primary databases used for the identification of studies for this research. The studies were identified using three sets of search terms (ST) (i = 3). The studies were initially identified from the databases and additionally from other sources such as Google scholar, government websites and references where applicable. All the studies were then screened to remove duplicates and to ensure that they were relevant to the research. The studies were also filtered out based on quality (quality of journal/conference, must be peer-reviewed). The number of studies (n_i_) obtained from each of these searches is denoted in Figure 1. There was no restriction enforced on the publication year since DM for CDSS, ED LOS and data quality are relatively older concepts still applicable in present context. This literature survey explores studies that use DM as part of CDSS in healthcare using studies selected from search i = 1. These studies were also used to determine the most appropriate key performance indicators for interpreting our results. We survey potential factors affecting LOS in Australian ED context using studies from i = 2. Since we acknowledge the significance of data quality on accuracy of results, we explored the impact of data quality in similar studies using literature from i = 3.

### 2.1. General Processes in Australian EDs

There is currently no standard around the world for EDs and processes such as triage vary from country to country [19]. Hence, for this study, we exclusively take into consideration the processes adopted in Australian EDs. There are three stages involved in the working of an ED [20]. The first stage involves the assessment of the patient and a high-level mapping resource that may be needed for that patient. At this stage, the patient is assigned a triage category (Resuscitation, Emergency, Urgent, Semi-urgent, or Non-urgent) by ED staff based on the acuity [7]. Following this, staff is scheduled, and finally, relevant internal departments are consulted, and further patient testing and assessments are conducted.

This final stage was found to take the longest time to complete, usually affecting ED LOS. There have been many efforts to streamline the ED process, for example introducing measures to reduce the amount of paperwork at each stage. However, there have been no efforts into reengineering the ED process and it has remained the same for many years. Although limited, there has been some research into how DM can be used as part of CDS to make the processes in EDs more efficient. Some instances of DM used as part of CDSS in EDs are predicting patient pathway, predicting patient admissions, pathology ordering, customer relationship management (CRM), and predicting ED LOS [21]. Several factors were found to increase ED LOS and are discussed in the following sections.

### 2.2. Data Mining in Predicting LOS

As a result of digitization, hospitals are collecting and generating large volumes of data each day. DM is now being used to extract value from these data to address challenges faced in the healthcare industry. DM is a methodology that transforms data into useful information primarily through relationship and pattern identification [20,21]. Building DMMs is one aspect of DM that involves summarizing large portions of data into more convenient forms. This process helps to uncover patterns and knowledge within data [22]. Many studies over the years successfully used DM in CDS. CDS provides healthcare professionals or patients with intelligently filtered clinical knowledge, patient information, or any other healthcare information at appropriate times to aid health-related decisions or actions [23]. Clinical Decision Support Systems (CDSS) are a class of information systems that aid clinical decision making. CDSS in some cases use artificial intelligence methods such as DM together with the patient or clinical data to assist in decision making [24]. Through the use of DMMs, we can create effective CDSS [25]. The use of DM in predicting patient LOS is still relatively unexplored when compared to other areas of healthcare and CDSS. Although there are studies that explored factors impacting patient LOS, the research into predicting these factors using DMMs is still limited. We are able to identify only two other studies to our knowledge that used DMMs to predict ED LOS in an Australian ED setting [4,6]. These studies both suggest that there is a potential to use DMMs to identify factors that impact ED LOS.

A class of DMMs called Predictive DMMs (PDMM) were found to be successful in predicting patient LOS [26,27]. PDMMs are primarily used for performing data classification and pattern matching tasks [28]. Currently, there is no specific method defined in the literature for PDMM selection. A PDMM can be selected based on several factors such as dataset size, type of data available, aims of the research, and expected outcomes. For example, the Random Forest (RF) algorithm was found to be a suitable PDMM to use on a large dataset [29]. PDMMs such as DTs, NB, K-NN, Artificial Neural Networks (ANN), and Gradient Boosting Machine (GBM) have been used previously in an LOS or ED LOS context [6,30,31,32]. Another factor that determines PDMM selection is the number and type of attributes being used for the study. These factors are discussed in the following section.

### 2.3. Factors Affecting Patient ED LOS

Factors such as patient age, gender, triage category, patient’s mode of arrival, needing an interpreter, admission, complexities in diagnosis leading to additional diagnostics, and availability of resources such as staff and beds are the most common previously identified factors that impact the ED LOS [4,5,6,33,34,35,36]. Many studies reported patient age to have an impact on ED LOS. It has been found that older patients were at a higher risk of having undiagnosed underlying medical conditions, often leading to an increase in diagnosis time and subsequently the LOS. These complexities in health conditions were also found to result in the prescription of additional pathology testing and scans for accurate diagnosis, and patients needing additional scans or pathology testing often experience delays in ED LOS [33,36,37]. It was also hypothesized that patients arriving by ambulance were more likely to be presenting with severe conditions compared to those arriving by other means of transport such as personal cars. This is why the arrival mode of a patient at the hospital could determine their ED LOS [2,36].

Triage category is another important factor that has been found to impact ED LOS [38]. All Australian EDs follow a standardized triage category system which ranges from 1–6 with decreasing level of patient severity. All patients are assigned a code by the triage nurse upon arrival at the ED based on their presenting condition. As a result of this, medical attention is given sooner to those patients who appeared to be presenting with more severe conditions (triage 1–3) than those with triage category above 3. This could mean that patients assigned a less critical triage might end up having to wait longer to be treated [36]. Time taken for doctors to first examine a patient and delays in diagnostics is indicative of ED crowding. This also could indicate the resource availability for that ED [34].

Some studies suggested that patient gender and ethnicity are also factors affecting ED LOS, even though there was no sufficient evidence to prove this [4,39,40]. For instance, it was found that ethnicity only impacted ED LOS in cases where there were language barriers or communication issues [41]. Despite this, both these factors were considered for this study. Other factors such as patient admission and requiring an interpreter were also found to impact ED LOS. This is because those requiring admission or interpreters might have to wait longer for diagnosis or scans due to severity in condition or complexities in communication, respectively [6,42]. Even though other factors such as patient insurance and number of staff available in the ED were found to impact ED LOS, they are considered to be out of scope for this study as they were not included in the dataset [6,37,43]. Many of these factors are either directly available in the VEMD or can be derived from VEMD attributes. These factors can be used as predictors to build PDMMs as detailed in the following sections.

## 3. Research Methodology

This section discusses the dataset used in this study along with an overview of the dataset quality. Following this, steps involved in the pre-processing and analysis of these dataset are discussed.

### 3.1. Dataset

A de-identified data extract containing all elements from VEMD plus additional administrative data were obtained from three locations of Healthcare Service A. Data collected at the three locations of Healthcare Service A for the year 2019 was obtained and used for this study. The data obtained for this study was collected before the COVID-19 pandemic and does not take into account factors such as shortage of resources or any other special circumstances.

### 3.2. Data Quality of the VEMD

Data quality is a crucial factor in DM and machine learning. Data that are deemed suitable for personal or office use might not be considered fit to be used in machine learning [44]. This is why data quality was considered to be a crucial part of this study. The overall data standard and quality of the VEMD is regulated by the Health Data Standards and Systems, Victoria. Each hospital using the VEMD is responsible for providing its staff adequate information and training regarding its usage. To ensure data accuracy, the VEMD is subject to frequent audits by the Victorian Agency for Health Information. Additionally, the validity, completeness, coherence, accessibility, timeliness, and interpretability are verified by health services [11].

Along with the standard VEMD attributes, the dataset obtained for this study had several additional attributes introduced by staff for internal uses. Many of these attributes are either irrelevant to this research or of low data quality. For example, an attribute called “presenting condition” had over 7000 unique values which were either misspellings or a variation of the same values. Including this attribute in the analysis may have been beneficial to the study by helping improve model utility and performance [6]. Despite this, due to its low quality, it was removed from the analysis. This issue is consistent with the findings from other studies that reported human-related data entry issues to be the biggest reason for quality issues of the VEMD [45]. Data quality ultimately determines the accuracy and reliability of a PDMM. Through exploration of the dataset and cleaning, the quality of data can be improved [46]. This pre-processing of the dataset to improve data quality is discussed further in Section 3.3.

### 3.3. Data Pre-Processing

The dataset for this study was created by merging VEMDs obtained from three locations of Healthcare Service A. There was a total of 92 attributes and 173,012 instances present across all three datasets combined for the year 2019. This dataset consisted of additional attributes (not specified in the VEMD documentation) added by the hospital staff for internal purposes. With the exception of one attribute called “mental health”, all additional attributes were found to be irrelevant to this research and were removed from the dataset. The “mental health” attribute indicates whether a patient was reviewed by a mental health professional, reviewed by the drug and alcohol unit, reviewed by both of these, or by neither of these. This information can be useful to understand if presentations requiring mental health or drug and alcohol assessments have a longer ED LOS [6].

To prepare the data for classification, a new “Class” attribute with values “>4” or “≤4” was added to the dataset. Of the total presentations, 38.62% (66,819) were found to have an ED LOS >4 h. This attribute was derived using the “ED LOS mins” attribute from the dataset. Additionally, since we also hypothesized that performing additional diagnostics might impact ED LOS, new attributes: “pathology needed?”; “MRI needed?”; “ultrasound needed?”; “CT needed?”; and “X-ray needed?” were introduced. An attribute called “doctor mins” was present in the dataset which captured the number of minutes it took for the doctor to first examine the patient. It was suggested that delays in doctor examinations could ultimately result in longer patient stays [47]. To capture this metric, a new attribute called “greater than average?” was added to capture instances where the doctor took greater than the average time to attend to a patient. The average time taken by a doctor, in this case, was 73.88 min with 35.47% of the instances being above the average. Here, the average time was calculated using the “doctor mins” column. Additionally, a new age group “≤18” was added to the “age category” attribute to categorize pediatric presentations and 7 instances in the dataset that had an invalid triage category of 0 were removed. The dataset size after the removal of these 7 instances was 173,005. Missing values were replaced with “nulls” and retained in the dataset for analysis. There were 4 missing values in “age category” which with corresponding “class >4”. Hence, these missing values were replaced with “nulls” and retained in the dataset. However, there was no significant improvement in model performance with the removal of these “nulls”. Finally, a Weka executable file with .arff extension was generated for further data pre-processing and analysis.

Pre-processing of data was performed using the “Explorer” tab on Weka [19]. Two attributes, “triage category” and “sex” were reclassified as nominal values. A total of 16 attributes either directly used from the VEMD or added to the dataset were used for analysis. These attributes are listed in Table 1.

### 3.4. Data Analysis

Classification of the data was performed using ZeroR, J48 DT, RF, LazyIBK, and NB models. The ZeroR model was used as a baseline classifier as it predicts the majority class and ignores all other predictors [48]. Additionally, since the dataset had categorical values, an LR model was also implemented [49,50]. Previous work has shown that performing data analysis on a single dataset led to biased or exaggerated model accuracies [51,52]. Although methods such as k-fold cross validation can help overcome this issue, splitting the dataset into training and test data was found to be more efficient especially on larger datasets [18,32,53,54]. Considering the size of the dataset available and benefits associated with splitting the dataset, this method was implemented during analysis.

For this study, 80% of the data were used for training and 20% for testing by using Weka’s Percentage Split (PS) feature. Data are randomly shuffled into either training or test subsets based on the “random seed” value specified in the model settings. To obtain the correct accuracy, the model was run 10 times with seed values ranging from 1 to 10. The average of model accuracies from each of these runs was adopted as the accuracy of that model [55,56].

**Figure 2:**
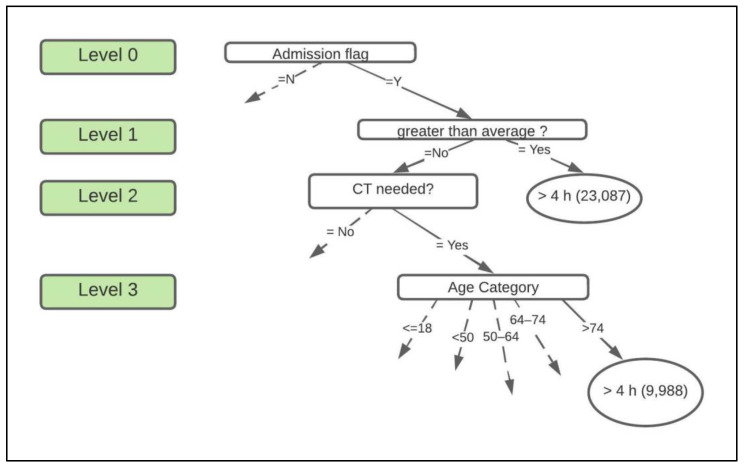
Portion of the decision tree. Note that the dotted lines indicate that the branch is connected to the rest of the tree. The ovals contain decisions, and the rounded rectangles contain the class.

First, the ZeroR algorithm was executed to obtain the baseline accuracy for the study. This baseline was used later to compare results from the other models. Next, the J48 algorithm was executed using the PS test option. Weka’s J48 DT algorithm produces a set of nodes, leaves, and branches that are created based on test cases. The outcomes/decisions for each of the tests are displayed as branches of the tree originating from nodes that hold the test cases. For instance, the tree tests “greater than average?” at the node and produces “Yes” or “No” branches along with the number of instances for each outcome or new branch (see Figure 2).

For this study, a pruned DT with a confidence factor of 0.25 was produced. There was no significant difference in model performance when the DT was left unpruned or reduced error pruning was used. Following this, the RF algorithm was implemented. RF models provide accurate results when bagging is performed as it is not prone to overfitting [29]. This is because the RF works by computing several small decision trees before producing one final decision. Hence, RF models can be run on the complete dataset, unlike DTs, which can overfit. The “bag size percent” value can be changed in RF model settings before execution. For this study, the default “bag size percent” value of 100 was used. Next, the K-NN model called the LazyIBK model on Weka was implemented. Similar to the RF, the K-NN algorithm does not require training [57,58]. A k-value is to be specified in the model settings before execution. Increasing or decreasing the k-value impacts the model performance. It was reported that larger k-values improved model performances for larger datasets [56,59]. For this study, a k-value of 1 was used. K values up to 50 in increments of 5 were tested, but these models had an insignificant change in accuracy. Following this, both the LR and NB models were implemented using the PS method. The results obtained from these models are presented in Section 4.

## 4. Results

A total of 173,005 ED presentations recorded by three locations of Healthcare Service A in the year 2019 were used in this study. Of these presentations, 38.62% reported having an ED LOS > 4 h, while 61.38% reported an ED LOS ≤ 4 h. The average time it took for a doctor to first examine a patient was 73.88 min. Six PDMMs were implemented for this study. Of these six models, the J48 DT, LR, NB, and ZeroR algorithms were implemented using the PS feature on Weka. The accuracy for these models was computed as the average accuracy obtained over 10 iterations with different seed values [55,56]. The ROC and number of correctly classified instances known as the accuracy of the model were adopted as the primary performance metrics in this study. They were identified to be suitable to be used in the ED LOS context [4,6].

The first model, ZeroR, had an average baseline accuracy of 61.41% and Standard Deviation (SD) of 0.154. The ROC value for this model was 0.05 out of the maximum value of 1. The next model, J48, had an average accuracy of 72.10%, SD of 0.1, and an ROC value of 0.762. This model’s accuracy and ROC are significantly higher than the baseline accuracy. The DT had a total of 944 leaves which were generated based on test cases. One part of this DT can be seen in Figure 2.

In this figure, we see that a total of 23,087 patients experienced an ED LOS >4 h when their first doctor visit lasted longer than the average time and were admitted into the inpatient ward. On the other hand, if the patient waited less than the average time to first be examined by a doctor but required a CT scan, the age of the patient determines their ED LOS. In this case, 9988 patients aged >74 stayed longer.

The NB model yielded an average accuracy of 70.23% with an SD of 0.167 and an ROC of 0.758. The LR model yielded an average accuracy 71.33% with an SD of 0.155 and ROC value of 0.773. Both these models had an accuracy and ROC value lower than the J48 model. However, they were still higher than the accuracy and ROC of ZeroR. Next, the LazyIBK model was implemented with k = 1. This model had an accuracy of 74.04%, along with an ROC value of 0.82. For the LazyIBK model, several k-values starting from k = 1 to k = 50 were tested in increments of 5. The model had an average accuracy of ≈72% when k values other than 1 were tested out. Finally, the RF model had an accuracy of 74.024% with an ROC value of 0.81. Both the RF and LazyIBK had the highest ROC and accuracies of all the models. The models implemented using PS did not show any significant change in accuracy when varying the seed values. This is why there is no significant difference in SD for these models. The f-measure, recall and precision of each of these models was also computed. The ZeroR model did not produce any recall or precision as it focuses only on the majority class, which is “≤4” in this study. The overall performance metrics form the data analysis can be found in Table 2.

## 5. Discussion

This study found that more than one-third of the ED presentations in the year 2019 had an ED LOS > 4 h. This was slightly higher than what was reported by other Australian EDs [4,6]. Of the six models that were implemented in this study, RF and LazyIBK models had the best model performance. The LazyIBK model had an ROC value of 0.82 and the RF had an ROC of 0.81. An ROC value of 1 indicates a perfect test with any values close to 0.7 considered to be acceptable. ROC values above 0.8 for medical research are considered to indicate excellent model performance [60]. The other three models, J48, LR, and NB had acceptable ROC curve values, while the ZeroR model could be considered an imperfect test. ROC values reported in other medical studies range from 0.6 to 0.86 [4,61,62]. The ROC values of both the RF and LazyIBK were consistent with what was reported by another study conducted in the Australian ED context [63].

LazyIBK, which is a Weka implementation of K-NN used in this study, had nearly the same accuracy of 74% as RF, while J48, DT, NB and LR performed with a slightly lower accuracy. The J48 DT, RF, and NB models had higher accuracies when compared to some studies in the LOS context, which reported accuracies around 63–72% using variations on classification [18,64]. There is only one other published study that used J48 DT to predict LOS in the Australian ED context [6]. This study reported an accuracy of 85% which is higher than our results. The inclusion of factors such as “presenting condition”, which were removed from this study due to data quality issues could be a reason contributing to a higher model accuracy in their study [63]. Consistent with this finding, model performance for RF and DT in this study had increased to around 83% and 82%, respectively, with the inclusion of the attribute “presenting condition”. Despite identifying this factor to be significant in determining ED LOS, it was not included in the final study analysis and results due to its poor quality. In our dataset, this attribute had nearly 7000 distinct values, many misspellings or redundancies which could not be cleaned without domain knowledge.

This study found that the performance of all the models improved after the data were pre-processed when compared to using the data in its original form. This confirms pre-processing data to improve its quality is an important step when implementing DMMs as suggested in literature [46]. The J48 DT implemented in this study was successful in identifying 944 possible outcomes based on the factors used in the study. This DT helped identify correlation between various factors used in analysis (see Figure 2 in Section 3.4).

Out of the 16 attributes used in the analysis, only six attributes were found to have a significant impact on patient ED LOS. These include patient age, time taken for the doctor to first see a patient, the patient mode of arrival, triage category, need for admission, and performing CT scans. This study found that patients aged between 64 and 74 and patients older than 74 were more likely to have an ED LOS greater than 4 h when compared to the others. This is consistent with other studies that suggested that older patients were likely to have a longer ED LOS [26,36,65]. The average time taken for a doctor to first see a patient was found to be 73.88 min. These results were consistent with the other Australian EDs, but higher than some outside Australia [7,66]. This research found that around 66.7% of the patients who were admitted into the hospital and waited longer than the average time to first be seen by a doctor had an ED LOS greater than 4 h. Additionally, those patients who had waited a greater than average time to first be seen by a doctor and also required a mental health review were found to have an ED LOS greater than 4 h. This is consistent with studies that suggested that patients who wait longer to be seen by a physician, require admission or require a mental health review experience longer ED LOS [6,47].

Studies also suggest that a longer waiting time to be seen by a doctor could be indicative of overcrowding [34,35,67,68]. Hospitals can customize these PDMMs to make critical decisions for reducing delays in diagnosis based on their present ED conditions. It was also found that around 43% of those who arrived by a mode of transport other than an ambulance, police vehicle, or community transport had an ED LOS which was less than or equal to 4 h. Those patients who arrived at the ED in any ambulance (air or road) had a higher probability of experiencing an ED LOS of greater than 4 h. This is consistent with findings from previous research [2,36].

Previous studies suggest that patients who required additional diagnostics were more likely to experience longer ED LOS [69,70]. This was found to be true only for patients requiring CT scans. This finding is consistent with other studies that suggest that CT scans often cause delays in ED LOS [37]. Other tests such as MRIs, ultrasounds, pathology, and X-rays were found to have the least impact on patient ED LOS out of all 16 factors. This is contradictory to other research, which found that MRI scans and pathology such as blood tests were equally responsible for delays in ED LOS as CT scans [69,71]. Based on the data used in this study, the number of CT scans that were performed were significantly higher than any other diagnostics tests. This could explain why the results from this study indicate only the connection of CT scans to delayed ED LOS compared to any other diagnostics. This may also confirm the hypothesis that CT scans are often over-prescribed in EDs [37,72].

Many studies also reported that patients with less severe triage codes (above 3) experience delays in diagnosis and treatment which impacted their LOS [6,47]. Contrary to this, our study found that more patients with triage category 3, who were aged between 50–64 and those aged >74 experienced ED LOS > 4 h compared to the rest. In the dataset used for this research, triage category 3 had a significantly higher number of patients than triage categories above 3 when ED LOS exceeded 4 h. Furthermore, the DT yielded an outcome of “>4” for triage category, mostly when patient age was considered. This is consistent with other studies that suggest that triage category impacts ED LOS based on the age of a patient [36].

The DT obtained in this study indicated a total of 944 possible outcomes and attribute relationships in the form of DT branches. Based on these relationships, it was also found that the admission of a patient into the hospital is classified based on factors such as age, triage category, and mode of arrival. This is why it was determined to have an impact on patient ED LOS. This is consistent with previous studies that considered age to determine patient admission and the delays in ED LOS due to these factors [6,63]. Along with confirming this, our study was also able to determine that factors such as triage category and mode of arrival to also determine patient admission and subsequent delays in ED LOS. Factors such as indigenous status, gender, preferred language, and needing an interpreter were found to have the least impact on ED LOS. Although most studies found language not to be significant, some still suggested that language barriers caused ED delays [41].

This study also confirmed that using the column “presenting condition” resulted in improved model performance as suggested by other studies [6,63]. We found data quality to be a significant factor in our research and believe that adopting measures to govern data quality would have a major impact on improving future research and the overall usability of data available [44]. One way to govern data to improve its quality is to standardize the information being collected. By establishing the purpose for collecting data could help decide rules around its collection and storage. Adopting these practices will be beneficial to not only hospitals but also to those using these data for research [73].

## 6. Conclusions and Recommendations

This study found that it is possible to predict patient ED LOS using factors impacting ED LOS as identified in studies. The PDMMs built in this study can offer useful insights into ED processes and also factors that risk delays in EDs. This information can be used by hospitals to monitor their EDs and make strategic decisions for improvement. Currently, there is limited research into the applications of DM in CDS. This study has shown that PDMMs can be used in CDS to help address challenges such as ED LOS. To our knowledge, only the J48 DT and GBM models were implemented in the Australian ED LOS context [4,6]. This study addresses the current lack of research in this context and shows that other PDMMs can also be used in predicting ED LOS. This study provides evidence that PDMMs such as RF, NB, LazyIBK, and LR can be implemented to address delays in LOS in Australian EDs. The results obtained in this study can offer insights for future research for both the applications of PDMMs in predicting ED LOS and the applications of DM in CDS. Future work can utilize machine learning and deep learning algorithms and frameworks for CDS and predicting ED LOS. This will help to determine predictive accuracy of more advanced models when compared to DM [74,75,76].

The DT obtained in this study was useful in identifying useful relationships between the various factors and their impact on the ED LOS. This study found that factors such as patient age, patient admission, the time taken to first be seen by a doctor, the arrival mode of the patient, requiring a CT scan, and triage category have the most impact on ED LOS. Those patients who waited longer than the average time to first be seen by a doctor and those who required a CT scan were at the greatest risk of experiencing ED LOS > 4 h. It was also found that those patients older than 64 years of age were at a higher risk of experiencing ED LOS > 4 h. This study also confirmed the correlation between patient age and admission and their role in causing delays in ED LOS. In addition to age, both triage category and arrival mode were found to be related to patient admission eventually resulting in delayed ED LOS. The DT also revealed that triage category had an impact on ED LOS based on other factors such as patient age. Additionally, the gender, indigenous status, or language of a patient were found to have the least impact on ED LOS. Contrary to other studies this study also found diagnostics like X-rays, pathology, MRI scans, and ultrasounds also had the least impact on ED LOS.

Studies also identified data quality to be an important factor when implementing a DMM. This study confirms data quality to be an important factor in data analysis. This study found that by pre-processing the data before analysis resulted in better model performance. Therefore, attributes such as “presenting condition” were excluded from this study. Future work can include such attributes as part of their data analysis. Future work could also investigate how these predictions vary based on hospital and data being used. This study was conducted in the Australian ED context. The models developed in this study need to be validated prior to international use, as ED processes differ around the world [77]. Customizing and using these models in other hospitals could vary the impact of certain factors such as diagnostics. Contrary to other research, this study found that only those needing CT scans were associated with ED LOS > 4 h, while other diagnostic tests had no impact on ED LOS. This finding could vary based on the hospital and resources available. The results in this study were reported as an average of 10 runs as recommended in the Weka documentation [56]. Future works could run experiments with the models a higher number of times and report on parameters such as outliers in the data.

This study was limited to the data readily available via the VEMD, with the addition of some other data points routinely collected by the industry partner. Factors such as vital signs, pain scores, along with social factors, insurance status and ED crowding could be incorporated into future research. This study also did not take into consideration the number of beds or staff in the ED. It was suggested that the number of staff working could impact ED LOS. This is because a shortage in staff and beds results in delayed patient diagnosis and testing [4,78,79]. Diagnostic results handover time was also found to be significant in increasing ED LOS. The handover time can be defined as the time taken for the diagnostician to handover the results of the test (for example, CT scan results) to the doctor [4]. Future work could include these factors as part of their research.

## Figures and Tables

**Table 1:** Summary of Attributes Included in Analysis.

Directly Used	Derived/Changed from the Original
sex	class
triage category	X-ray needed?
indigenous status description	pathology needed?
interpreter require description	CT needed?
preferred language	MRI needed?
arrival mode description	ultrasound needed?
mental health	greater than average?
admission flag	age category

**Table 2:** Summary of the Performance Metrics.

Measure	J48	LazyIBK	LR	NB	RF	ZeroR
Accuracy	72.10%	74.04%	71.33%	70.23%	74.024%	61.41%
ROC	0.762	0.82	0.773	0.758	0.81	0.05
F-Measure	0.716	0.735	0.706	0.699	0.736	-
Recall	0.72	0.74	0.713	0.701	0.74	0.613
Precision	0.716	0.736	0.707	0.698	0.736	-

## Data Availability

Restrictions apply to the availability of these data. Data was obtained from Healthcare Service A and are available from the authors with the permission of Healthcare Service A.
